# Activation of PARP2/ARTD2 by DNA damage induces conformational changes relieving enzyme autoinhibition

**DOI:** 10.1038/s41467-021-23800-x

**Published:** 2021-06-09

**Authors:** Ezeogo Obaji, Mirko M. Maksimainen, Albert Galera-Prat, Lari Lehtiö

**Affiliations:** Faculty of Biochemistry and Molecular Medicine & Biocenter Oulu, University of Oulu, Oulu, Finland

**Keywords:** Enzyme mechanisms, DNA damage and repair, PolyADP-ribosylation, X-ray crystallography

## Abstract

Human PARP2/ARTD2 is an ADP-ribosyltransferase which, when activated by 5′-phosphorylated DNA ends, catalyses poly-ADP-ribosylation of itself, other proteins and DNA. In this study, a crystal structure of PARP2 in complex with an activating 5′-phosphorylated DNA shows that the WGR domain bridges the dsDNA gap and joins the DNA ends. This DNA binding results in major conformational changes, including reorganization of helical fragments, in the PARP2 regulatory domain. A comparison of PARP1 and PARP2 crystal structures reveals how binding to a DNA damage site leads to formation of a catalytically competent conformation. In this conformation, PARP2 is capable of binding substrate NAD^+^ and histone PARylation factor 1 that changes PARP2 residue specificity from glutamate to serine when initiating DNA repair processes. The structure also reveals how the conformational changes in the autoinhibitory regulatory domain would promote the flexibility needed by the enzyme to reach the target macromolecule for ADP-ribosylation.

## Abstract (web summary)

Poly(ADP-ribose) polymerase 2 (PARP2) is activated by 5′-phosphorylated DNA breaks but the molecular mechanism is not fully understood. Here, the authors report a crystal structure of PARP2 bound to an activating DNA fragment, providing insights into the structural changes that lead to PARP2 activation.

## Introduction

DNA damage is a common event in cells; approximately 10^4^−10^5^ DNA lesions are experienced by the cells per day^1,2^. In human, proteins involved in DNA lesion site detection include enzymes of the ADP-ribosyltransferase family (PARP1–3/ARTD1–3)^1,3^. In the nucleus, the main enzymes carrying out poly-ADP-ribosylation are poly(ADP-ribose) polymerase 1 (PARP1) and PARP2. These enzymes bind to the damaged DNA and subsequently generate poly-ADP-ribose (PAR) chains that act as recruitment signals for a range of DNA repair factors^4–6^. The role of poly-ADP-ribosylation is established in single-strand break repair (SSBR) and in alternative non-homologous end joining (aNHEJ) mechanisms, where the key proteins involved in the repair pathways are known to be recruited to the site of DNA damage in a PAR-dependent manner^7–12^. Poly-ADP-ribosylation also initiates chromatin remodelling through PAR binding ALC1 (amplified in liver cancer 1)^3,13^.

PARP1–3 enzymes have a similar domain organization in the C-terminal catalytic region consisting of an ADP-ribosyltransferase domain, a regulatory domain (RD)^14^ and a WGR domain shown to participate in DNA binding^15–21^. However, they differ in their N-terminal parts as PARP1 contains a BRCA1 C Terminus domain (BRCT) and three zinc-fingers (ZnFs), whereas the N-termini of PARP2 and 3 are intrinsically disordered^17,22^. The WGR domain of PARP2 has been shown to be key to DNA damage recognition^17,18,22^. In vitro, PARP1 can be activated by multiple forms of DNA damage-mimicking oligonucleotides^23,24^, while PARP2 and PARP3 are specifically activated by 5′-phosphorylated DNA breaks^16–18^. Although PARP1–3 employ different mechanisms in DNA damage recognition, it is thought that their activation will be similar as they all contain an autoinhibitory RD domain. The RD domain, in the inactive state, covers the active site and prevents binding of the substrate NAD^+^ to the catalytic domain^24,25^.

Multiple structures of the catalytic domain of PARPs^26–29^ and of individual domains binding to DNA are available for PARP1 and PARP2^15,18,30–32^. For PARP1 activation, a conformational change at the catalytic fragment is necessary^24,25^ and that is triggered by the sequential reorganization of the protein domains on the detected DNA damage site^15^. Furthermore, binding of histone PARylation factor 1 (HPF1) to the PARP domain results in a joint catalytic site with changed specificity from glutamate and aspartate to serine^33,34^. Despite the recently reported structures and HXMS studies, it is still poorly understood how the binding of the enzyme to an activating DNA molecule can trigger a robust activity increase up to 500-fold^8,15,17,18^. Here we elucidate this process by describing the structural basis of PARP2 DNA-dependent activation. Activation induces major conformational changes in domain structure and reordering of the secondary structure elements of the RD domain to release the enzyme from an autoinhibited state.

## Results

### Crystal structure of PARP2-DNA

**Fig. 1:**
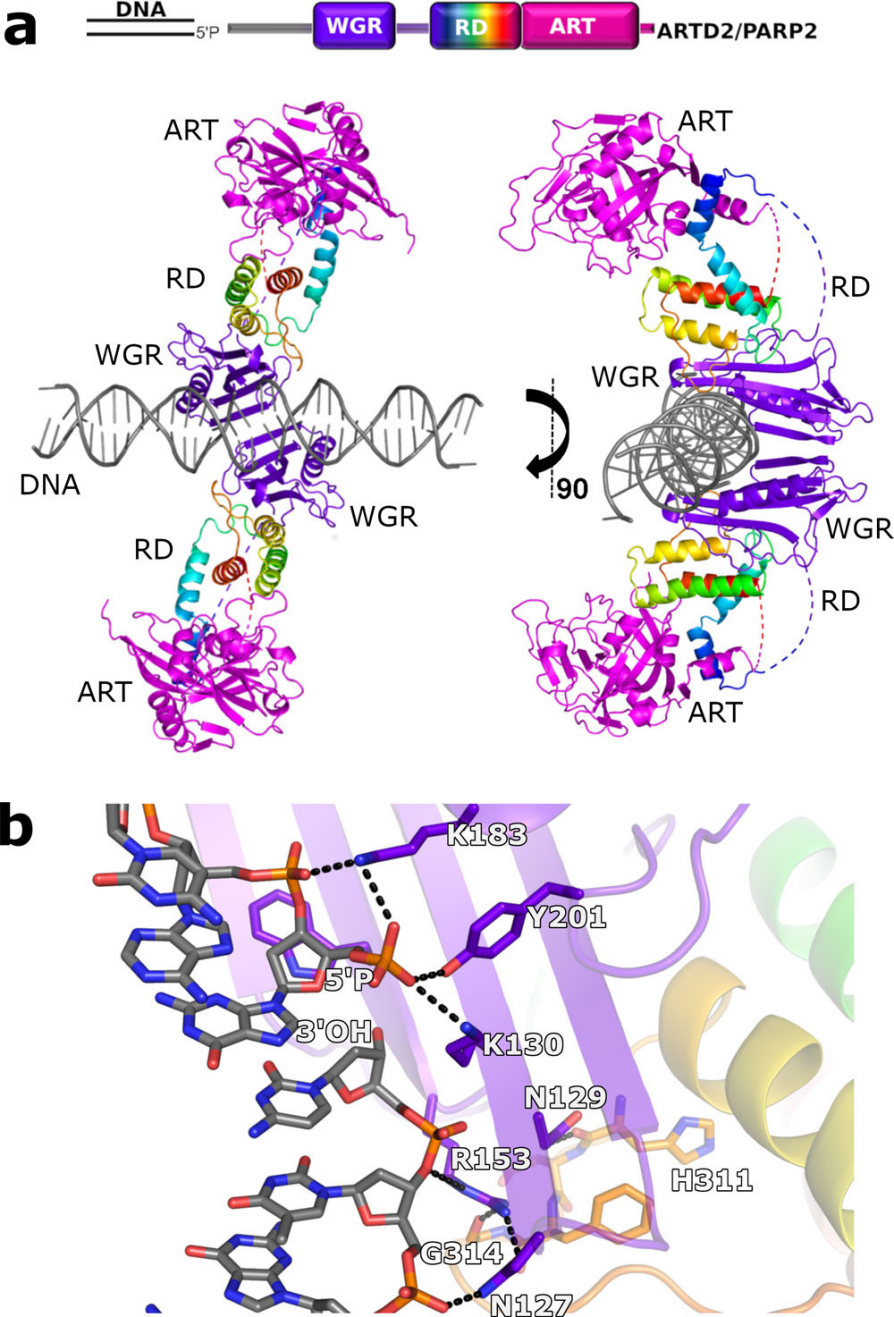
Crystal structure of PARP2 activated by binding to the DNA damage. **a** The biological unit as observed in the solution contains two dsDNA molecules joined together by two PARP2 molecules binding to the two nicks formed at the DNA break. The WGR domain is shown in purple, C-terminal transferase domain in magenta and RD from blue to red from N-terminus to C-terminus. **b** WGR domain and DNA interaction interface. Hydrogen bonds are shown as dashed lines. .

We determined a PARP2 crystal structure consisting of the WGR domain and catalytic fragment (PARP2_WGR-RD-ART_; residues 90-583) in complex with an activating double-stranded oligonucleotide mimicking a damaged DNA. The structure was obtained by using a multicrystal approach where 26 small datasets were merged in order to achieve a complete dataset at 2.8 Å resolution (Table 1 and Supplementary Fig. 1). The asymmetric unit contains one PARP2_WGR-RD-ART_ molecule with one dsDNA (DNA-1, Supplementary Table 1), while the biological unit has an apparent 2:2 stoichiometry in solution (Supplementary Fig. 2). The complex is formed by two dsDNA oligonucleotides joined together at the phosphorylated ends by two flanking proteins (Fig. 1a). The WGR domain of PARP2 interacts with the 5′-phosphorylated site and with DNA on both sides of the nick as observed with the isolated WGR domain^18^. The two DNA molecules and two PARP2s are related through a 2-fold symmetry axis. While two PARP2 enzymes are needed for efficient bridging of the double stand break, PARP2 is robustly activated also by a 5′-phosphorylated single-strand break. Due to this and the crystallographic symmetry defining the two PARP2 proteins of the biological unit to be identical, in the following we will consider a single PARP2 as a monomeric protein detecting a DNA nick.

**Table 1:** Data collection and structure refinement statistics for the PARP2_WGRCAT_ + 5′P-dsDNA complex structure.

Data collection	
PDB code	7AEO
Crystals	4
Datasets	26
Space group	I4_1_22
Cell dimensions	
*a*, *b*, *c* (Å)	166.83 166.83 143.84
α, β, γ (°)	90, 90, 90
Wavelength (Å)	0.9687
Resolution (Å)	50–2.8 [3.04–2.95] (2.87–2.8)
*R*_merge_ (%)	11.1 [80.2] (143.9)
*R*_meas_ (%)	12 [86.5] (156.0)
I/σI	9.25 [2.84] (1.6)
CC_1/2_	99.2 [79.1] (44.1)
Completeness (%)	98.9 [99.7] (99.6)
Redundancy	6.8 [7.0] (6.7)

Refinement	
No. reflections	24985
*R*_work_ / *R*_free_	22.7 / 27.1
No. atoms	
Protein	7120
DNA and ion	1024
Average B-factors (Å2)	142.96
Protein	143.96
DNA and ion	123.91
RMSD	
Bond lengths (Å)	0.005
Bond angles (°)	0.74
Ramachandran plot (%)	
Favoured	96.67
Allowed	7.78
Outlier	0.00

Values in parentheses are for the highest resolution shell.

CC_1/2_ defines correlation between mean intensities calculated from random half-sets.

*R* = ∑_*hkl*_ [*F*_obs_—*kF*_calc_] / ∑_*hkl*_ [*F*_obs_] for reflections used in refinement. *R*_free_ is *R* for 5% of reflections excluded from crystallographic refinement.

*R*_merge_ = (SUM(ABS(*I*(*h*,*i*)−*I*(*h*)))/ (SUM(*I*(*h*,*i*))) is the mean intensity of *h* observations of reflection *hkl* and its symmetry equivalents.

The WGR domain detects the phosphorylated DNA end coordinated by Trp151, Lys130, Lys183 and Tyr201. Tyr201 (Phe638 in PARP1) forms a hydrogen bond with the phosphate, whereas Arg153 and Asn127 (Gly565 in PARP1) interact with the N-1 nucleotide from the 3′ end and bridge the connection to Gly314 of the RD domain (Fig. 1b). The orientation of the residues is similar to the DNA complexes of the isolated WGR domain^18^ and to the recently reported cryo-EM structure^20^.

### Opening of the DNA end and interaction with the PARP2 catalytic domain

**Fig. 2:**
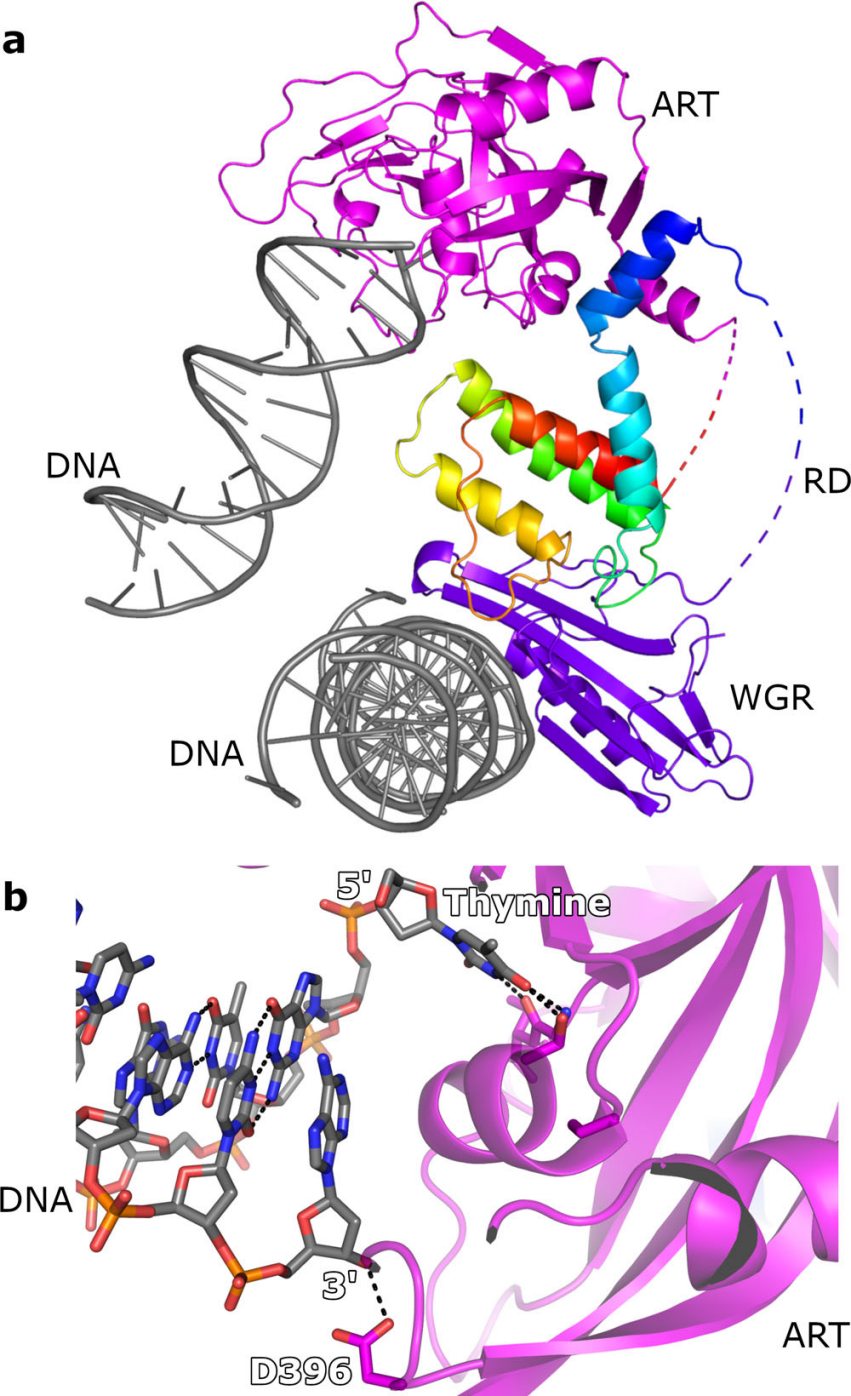
Binding of unphosphorylated DNA end to the catalytic site. **a** DNA end binding to the catalytic domain and formation of a crystal contact. **b** Interaction of the DNA 3′- and 5′-ends with the transferase domain. .

Interestingly, in addition to binding to the phosphorylated DNA break by the WGR domains, the crystal symmetry showed that the catalytic site was also in contact with the DNA, namely the unphosphorylated end, where the last A-T base pair is opened up (Fig. 1a). The 3′-adenosine ribose interacts with the helix lining the active site and its ribose makes only one hydrogen bond to Asp396, while the 5′-thymine binds to the active site (Fig. 2a) and locates in the nicotinamide binding site between two tyrosine side chains (Fig. 2b). The base forms typical hydrogen bonds with Gly429 and Ser470 like the amide group of the PARP inhibitors and the substrate-mimicking analog (Supplementary Fig. 3). In addition, the ribose and phosphate of the nucleotide bind to the same regions in the active site where the substrate NAD^+^ is expected to interact. Therefore, we performed an inhibition assay using thymine, thymidine and thymidine monophosphate (TMP) and the result showed that they indeed inhibited PARP2 with an IC_50_ of 50, 14 and 681 µM, respectively (Supplementary Fig. 4).

In addition, to determine how the activation capacity of the particular DNA used in the crystallization would affect the PAR synthesis of PARP2, we measured the DNA-dependent activity of the enzyme using the same DNA as in the crystal structure as well as other model DNAs. Indeed, the DNA used in the crystallization showed reduced PAR synthesis compared to nicked hairpin DNA and a longer form of dumbbell hairpin DNA (Supplementary Fig. 4). Conversely, when we substituted the thymine in the DNA used for the crystallization with guanine and used DNA of different lengths (DNA-4 and DNA-5) NAD+ hydrolysis increased (Supplementary Fig. 4). This indicates that a terminal A-T pair of the DNA would also inhibit PARP2 in solution.

### Local unfolding of the PARP2 RD domain and autoinhibitory effect

**Fig. 3:**
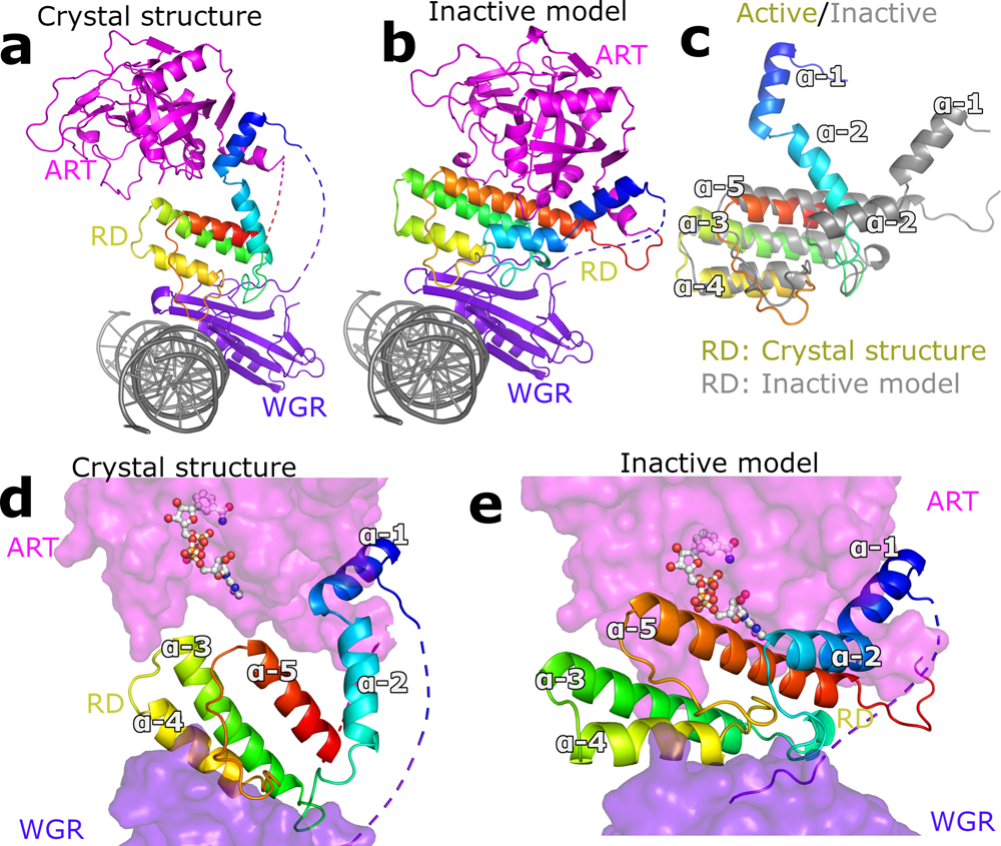
Conformational changes upon PARP2 activation. **a** Activated PARP2 structure. **b** A model of PARP2 binding to DNA in an inactive conformation where the PARP2_RD-CAT_ crystal structure (PDB id. 4TVJ)^36^ was positioned as observed on the PARP1 structure (PDB id. 4DQY)^15^. **c** Close up view comparing the RD domain in the active and inactive conformations. **d** Illustration showing how DNA activation releases autoinhibition of the catalytic domain allowing substrate binding (BAD shown in sticks, PDB id. 6BHV)^25^. **e** RD in an inactive state blocks the binding of the substrate NAD^+^.

Comparison of the PARP2_WGR-RD-ART_ structure with individual domain structures and the PARP1 DNA complex reveals major conformational changes that occur upon DNA binding. These include a movement of the ART domain with respect to the rest of the protein and reorganization of the RD domain. The RD reorganization leads to opening of the catalytic site and local unfolding, especially of helix α5 which packs against the catalytic domain in the inactive state (Fig. 3a–c). This is in line with previous studies using HXMS that show local conformational changes are required to unfold the RD helix as this inhibits enzymatic activity by covering the NAD^+^ binding site^23–25^. In addition, the crystal structure revealed that, while the RD region close to the DNA and interacting with the WGR domain remains unaffected, the conformational changes involve more than the unfolding of a helical fragment as both the N- and C-terminal helices of the RD region undergo major reorganization (Fig. 3a–c). Upon DNA binding, helix α5, covering the active site in the inactive conformation, is divided into two parts at Gly338, and the helices are completely reorganized. Subsequently, the catalytic transferase domain moves ~11 Å away from the DNA and it is rotated and translated implying mobility in solution (Fig. 3a-c).

### Mechanism of substrate binding by PARP2 upon DNA activation

Recently, a crystal structure of a constitutively active transferase fragment consisting of only a ART domain was solved in complex with an unhydrolysable substrate analog benzamide adenine dinucleotide (BAD)^25^. This PARP1 crystal structure was used to model substrate binding in PARP2 and compare its accessibility in the inactive and active conformations. The PARP2 inactive model is not compatible with substrate binding due to steric effects caused by RD residues (Fig. 3e). In the active conformation, the RD helices have moved and exposed the active site of the transferase domain, which is now fully capable of binding the substrate NAD^+^ (Fig. 3d). PARP2 is known to catalyse automodification and ADP-ribosylate itself in *cis* or in *trans*, the latter of which resembles modification of other proteins localized to the DNA lesion. PARP2 is also able to ADP-ribosylate the ends of the same dsDNA molecule where it is bound. This happens preferentially within a DNA molecule where the end is approximately 30 Å from the WGR binding site^35^. This indicates that the automodification of PARP2 or PARylation of DNA may happen in *cis* and that the transferase domain is indeed mobile when the enzyme is activated.

**Fig. 4:**
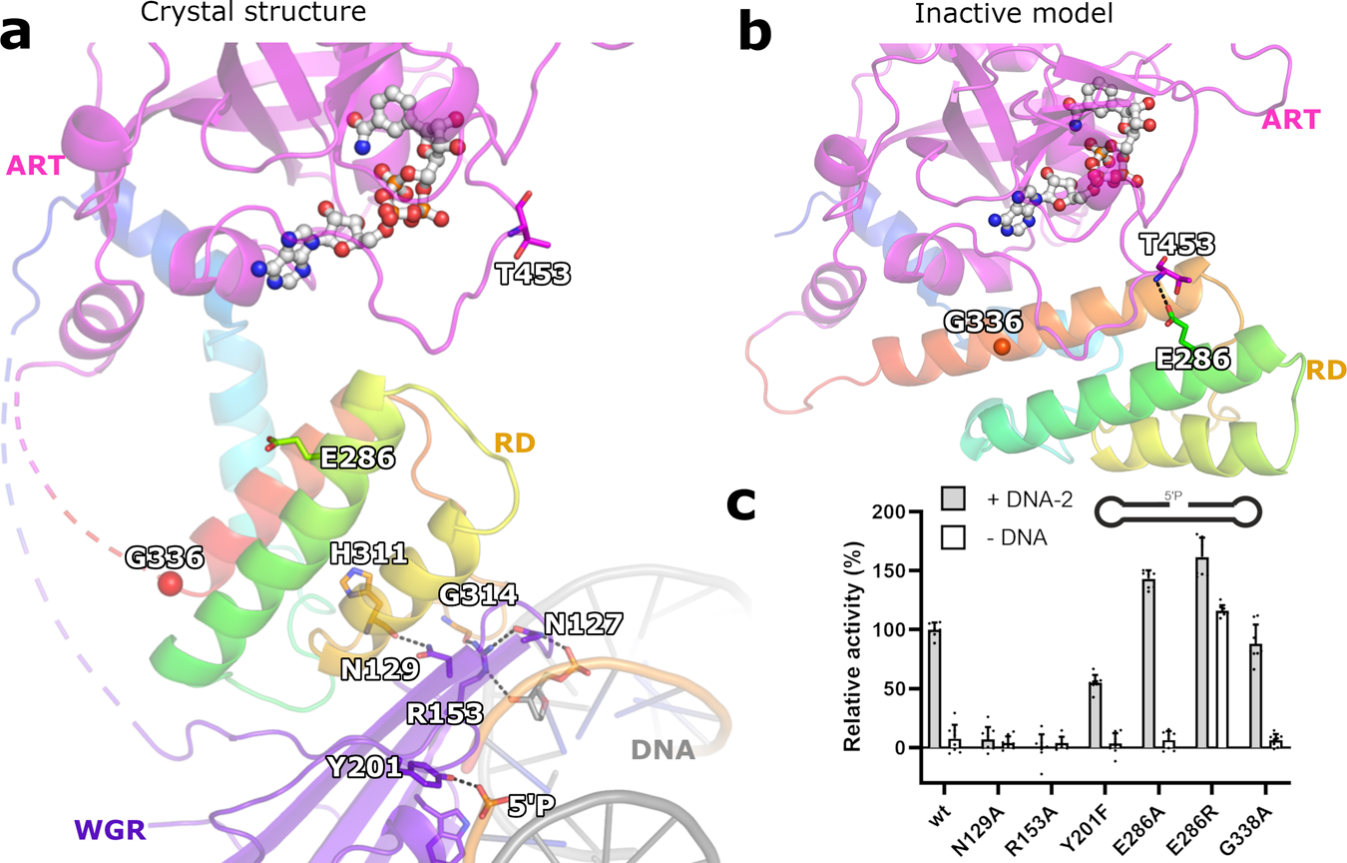
PARP2 activation mechanism. **a** Upon DNA binding the interactions formed by Arg153, Asn127 and Asn129 transmit the signal for the conformational change to release the RD–ART interaction. **b** A model of a PARP2 binding to DNA in an inactive conformation showing the interaction between the RD and ART domains in the inactive conformation. **c** DNA-dependent activity assay measuring NAD^+^ consumption (used at 5 µM concentration) after incubation with PARP2 enzymes (50 nM). Points correspond to conversion of 8 individual data points obtained from 2 independent experiments. Bars correspond to average and error bars to SD. Source data are provided as a Source Data file.

Asn129 (PARP1^Asn567^, PARP3^Asn79^), located between the WGR and catalytic domains, has been mapped as a key element in transferring the activation signal to the catalytic fragment^16^. We also confirmed that N129A is inactive (Fig. 4) although it retains the same nM affinity for DNA (Supplementary Fig. 5) and is properly folded (Supplementary Fig. 6). The adjacent residues, Arg153 and Asn127, also provide a link between DNA and the WGR and RD domains (Fig. 4a). We have shown that mutations in these residues result in loss of DNA-dependent activity and specific DNA binding (Fig. 4c)^18^. The DNA affinity of the full-length protein is driven largely by the disordered N-terminus, and the affinities of all the mutants generated in this study, including the above, also show similar low-nM affinity for nicked phosphorylated DNA and 18–150 nM K_D_ values for a dsDNA model (Supplementary Fig. 5)^17,18^. Arg153 and Asn127 interact with DNA at the 3′ side of the nick, with each other, and with a carbonyl group of Gly314 of the RD (Fig. 4a). Together with the Asn129 interaction, changes in these residues could result in the unfolding of some of the RD helices leading to release of the RD domain from the ART domain. Tyr201 is also important for the DNA binding of PARP2^16,18^ and critical for the nicked DNA detection by PARP2, as a Y201F mutation resulted in a 50% reduction of the catalytic activity measured by NAD^+^ consumption (Fig. 4c).

To further examine the changes observed for domain interactions we designed key mutations at the RD–ART interface. We observed that the RD and ART domains have contacts at Glu286 of the RD and Thr435 backbone amides and hydroxyl located at the D-loop lining the NAD^+^ binding cleft in the inactive state (Fig. 4b). We rationalized that by disrupting these contacts we could create an enzyme, which would be active even in the absence of DNA. Mutant E286A showed slightly increased activity in the presence of an activating DNA oligonucleotide, but only basal level activity in the absence of the DNA (Fig. 4c**)**. However, when reversing the charge with an incompatible E286R mutation we generated repulsion between the domains resulting in a hyperactive enzyme. The activity of the mutant was further increased by supplementing DNA indicating that the equilibrium clearly favours the active state more than the inactive state. Our effort to stabilize the helix α5 with a G338A mutation did not prevent activation (Fig. 4c) suggesting that a similar activation mechanism may also be possible in PARPs which lack the glycine in the same position (PARP1^Q717^, PARP3^E237^).

### Distinct modes of PARP2 automodification in the presence of HPF1

DNA binding has been shown to be prerequisite for binding of HPF1 to the ART domain, as the RD domain of PARP1 or PARP2 would prevent this in the inactive conformation^21,33^. Based on the ART and HPF1 complex structure (PDB code, 6TX3)^33^, we modelled the position of HPF1 to the activated PARP2. Our observed conformational changes in the PARP2_WGR-RD-ART_ DNA complex structure indeed allow binding of HPF1 to ART (Supplementary Fig. 7). This explains how, upon activation by DNA damage, the release of the ART domain allows the docking of HPF1 E284 to the catalytic core of PARP2 modulating the specific serine ADP-ribosylation^33^. It should be noted here that in the recent cryo-EM structure HPF1 binds to the closed form of PARP2^20^ and therefore the changes enabling HPF1 binding may be more subtle.

**Fig. 5:**
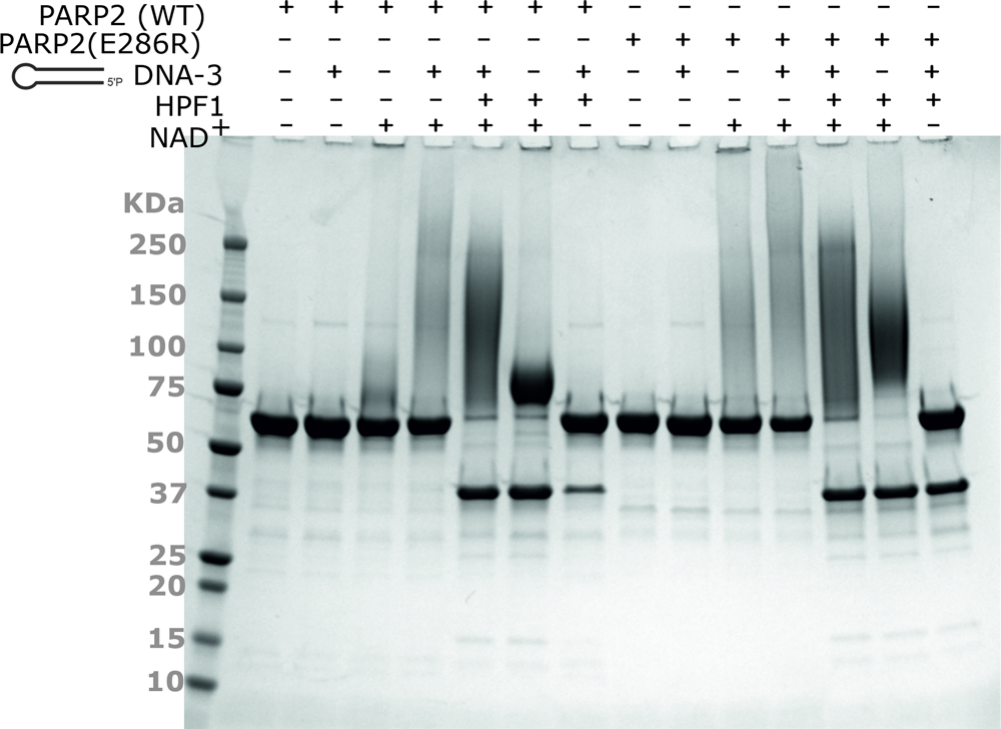
PARP2 activity assay with HPF1. Binding of HPF1 changes the nature of the resulting PAR. Reactions contained 5 µg PARP2 and equimolar concentrations of HPF1 and 5′ phosphorylated hairpin oligonucleotide as well as 1 mM NAD^+^ to allow visualization of protein smearing due to poly-ADP-ribosylation. The experiment was repeated with similar results.

We next tested the effect of HPF1 binding in a PARP2 automodification assay (Fig. 5). In the presence of NAD^+^, PARP2 automodification appears as a smear on an SDS-PAGE gel due to heterogeneous auto-poly-ADP-ribosylation (Fig. 5). The smear is more prominent when the activating DNA-3 is present and also in the E286R mutant, both with and without DNA, in agreement with the NAD^+^ consumption assay results. The addition of HPF1 drastically affects the behaviour of both WT and E286R by promoting automodification of apparently all PARP2 enzymes present (Fig. 5). The addition of HPF1 in the reaction resulted in a focused smear centred around 125 kDa. In the absence of activating DNA, HPF1 still promotes partial automodification of PARP2 resulting in a band shift of PARP2. Under these conditions, the WT PARP2 automodification is more homogeneous and the protein migrates as a band centred around 75 kDa, while E286R produces species centred above 100 kDa, similar to those produced in the presence of DNA. Altogether, this suggests that HPF1 potentially contributes to the activation of PARP2 through a mechanism involving disruption of the autoinhibitory effect of the RD domain.

## Discussion

PARP2 is a DNA repair enzyme and its catalytic activity is highly elevated in response to cellular genotoxic stress. Here we describe the structural mechanism of PARP2 DNA damage detection and mechanism of its activation upon binding to an activating DNA molecule. The current view is that the three DNA-dependent PARPs, PARP1–3, share similar activation mechanisms despite the distinct DNA recognition modes resulting from the differences in their domain organization^16^. The DNA binding mode of PARP2 has been shown to differ from that of PARP1, which uses the zinc finger and WGR domains to coordinate its binding to a DNA end, while PARP2 uses its WGR domain to create DNA end-to-end binding^18,20,21^. In contrast to PARP1, the in vitro activity of PARP2 is rapidly elevated only in the presence of 5′-phosphorylated DNA^16,17^.

**Fig. 6:**
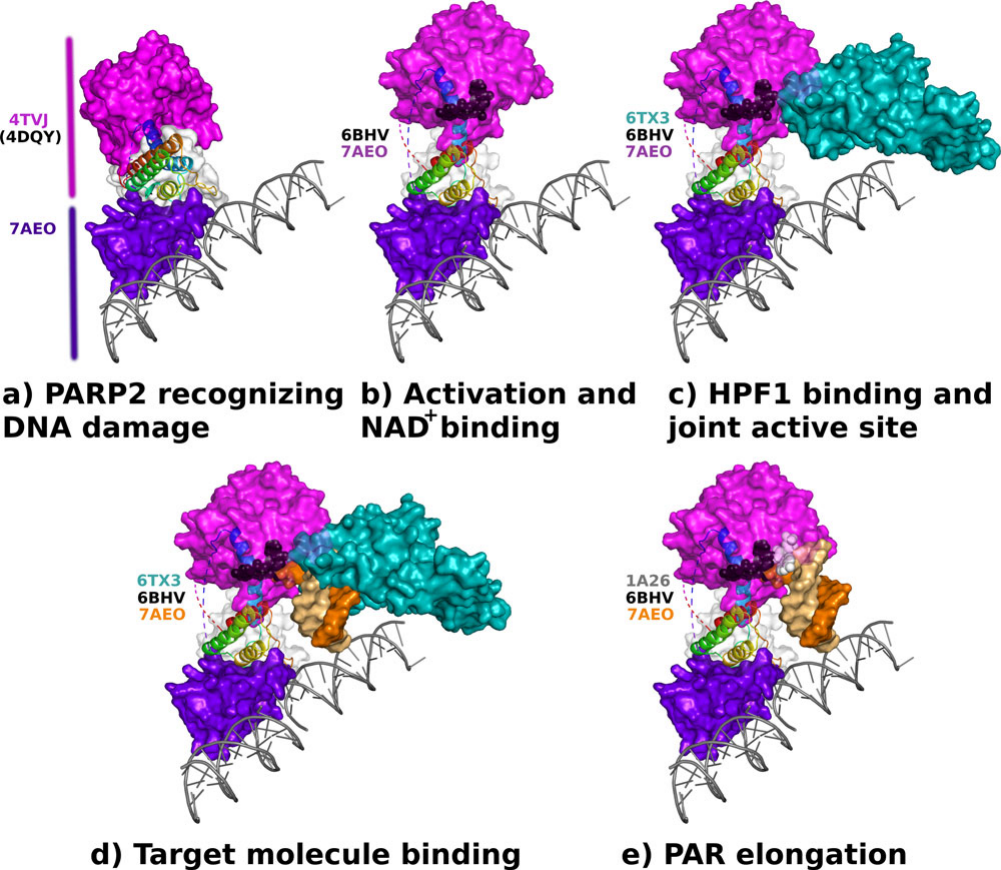
Conformational changes upon PARP2 activation and substrate protein modification. **a** Inactive conformation of PARP2 when bound to DNA. **b** PARP2 undergoes a conformational change in the regulatory domain α-helices allowing NAD^+^ (black) binding. **c** HPF1 can bind to the PARP2 catalytic domain to form a joint active site. **d** Conformational changes in the regulatory domain release the catalytic domain and allow it to modify target macromolecules marked here by the second DNA molecule observed in the crystal structure. **e** PARP2 can catalyse the PAR chain elongation reaction also without HPF1, which may dissociate from the complex. Carba-NAD^+^ bound to the acceptor site is shown in white. PDB codes used for the illustrations are indicated in the panels.

The crystal structure described here contains an almost full-length PARP2 lacking only the disordered N-terminal part responsible for nuclear localization and increasing unspecific DNA affinity due to its positive charge. The structure allows visualization of the series of events that enable PARP2 to bind the substrate NAD^+^ upon detection of a DNA damage site. It explains previous observations of local unfolding of the RD domain, reorganization required for HPF1 binding and how the catalytic domain is released from the inhibited state to allow modification of target proteins. A summary of the process, which is illustrated in Fig. 6 and in the Supplementary Movie 1, can be divided into five stages marked (a)–(e).

(a) Initially the binding of PARP2 to DNA is facilitated by the positively charged and disordered N-terminus, and when PARP2 recognizes a nick in the DNA it is locked in place by bridging the DNA gap with the WGR domain^18^. The structural conformation observed for the isolated catalytic domain is very similar to the PARP1^15^ structure in complex with DNA. As the structure is not fully compatible with binding of substrate NAD^+^, we used it as a template for superimposition of the PARP2 RD–ART fragment structure^36^. (b) PARP2 binding to 5′-phosphorylated DNA induces major conformational changes in the RD domain. Previously local unfolding and exposure of these elements was demonstrated by HDX-MS and by a low-resolution cryo-EM structure^20,24^. The conformational change allows efficient binding of substrate NAD^+^^25^. Crystal structures of the catalytic fragment have formed a basis for structure-based drug development efforts. Recently the difference in the PARP trapping efficiency of clinical inhibitors competing with NAD^+^ binding was rationalized to result from interactions with the RD^23^. The conformational changes we observed upon DNA binding could facilitate also the development of improved drugs. (c) When HPF1 binds to the catalytic domain of an activated PARP2, they form a joint active site and change the PARP2 residue specificity from glutamate/aspartate to serine^33^. Notably, the order of stages (b) and (c) is not established, as it was shown that a NAD^+^-mimicking inhibitor was required to establish stable binding of HPF1^33^, while this was not required in the cryo-EM structure^20^. In the cryo-EM structure, the large conformational change was not observed, but in that particular case, a cluster of positively charged surface residues of HPF1 interact with the major groove of the nearby nucleosome DNA likely limiting the conformational flexibility. Based on our experiments, the initiation reaction in the context of automodification is very robust when HPF1 is present as all the PARP2 proteins are modified (Fig. 5). (d) The large conformational changes in the RD allow binding of substrate macromolecules that get ADP-ribosylated. In our model, a substrate protein site is marked by the second DNA molecule observed in the crystal structure, which is bound to the active site by a thymine base. Some destabilization of the HPF1 helices was reported in the low-resolution cryo-EM data also for the HPF1 and this could enable substrate binding to avoid clashes with the proteins^20^. (e) PARP2 is able to catalyse the polymer formation alone as observed in vitro (Fig. 5). The so-called acceptor site has been mapped based on the crystal structure of the PARP1 catalytic domain with carba-NAD^+ 37^. The electron density for the substrate analog is not good and subsequently it was only partially modelled in the structure. Carba-NAD^+^ is bound to a location overlapping with the HPF1 binding site. This and the long polymers generated in the absence of HPF1 indicate that HPF1 can dissociate at some stage from the complex and allow robust generation of long PAR chains, as HPF1 binding is sterically not compatible with the generation of long polymers^33^. In Fig. 6, the second DNA molecule marks the target protein position in the elongation reaction, but it is not known what the exact site of the mono-ADP-ribosylated protein is at this stage. When the polymer is elongated it likely dissociates also from the PARP2. The sequence of these final events are not yet completely validated by experiments, and so far substrate proteins have not been observed bound to the activated PARP2 or other enzymes of the family. Our crystal structure of the open PARP2 structure, however, allows visualization of these events based on current knowledge.

## Methods

### Cloning, protein expression and purification

The cloning of the DNA constructs coding for PARP2_FL_ isoform 1 (residues 1-583: UniProt ID Q9UGN5) and the individual domain construct (PARP2_WGR-RD-ART_; residues 90-583) has been previously described^17,18^. Human HPF1 was cloned into pNH-TrxT and the resulting protein after tag cleavage contains an additional serine at the N-terminus. Mutagenesis of the PARP2_FL_ enzyme and PARP2_WGR-RD-ART_ were done using Quick change site-directed mutagenesis except for G338A, E286A and E286R mutants that were obtained by assembly PCR (Supplementary Table 2). Briefly, for each mutant, two fragments were PCR amplified, the first comprising the 5′ end until the mutation site and a second product comprising the mutation site until the 3′ end. The two fragments were then assembled by PCR and cloned using SLIC into pNIC-Zbasic or pNH-TrxT. All clones were sequenced using the automated sequencer in the Biocenter Oulu core facility, University of Oulu, Finland. Expression and purification of proteins were done as described previously^17,18^.

All proteins were expressed in *Escherichia coli* BL21 (DE3) using terrific broth autoinduction media (Formedium) supplemented with 8 g/l glycerol and 50 μg/ml kanamycin. For PARP2 constructs 10 mM benzamide was added to the media. The cells were grown at 37 °C with shaking until the OD600 reached 1 and then the temperature was lowered to 18 °C for 16 h or 15 °C for 24 h for PARP2 E285A, E286R and G338A mutants. The cells were harvested by centrifugation (4000*g*, at 4 °C for 30 min) and suspended in lysis buffer [50 mM Hepes (4-(2-hydroxyethyl)-1-piperazineethanesulfate), 500 mM NaCl, 10% glycerol, 10 mM imidazole, 0.5 mM TCEP (*tris*(2-carboxyethyl)phosphine), pH 7.5] and supplemented with 0.1 mM Pefabloc (4-(2-Aminoethyl) benzenesulfonyl fluoride hydrochloride (Sigma-Aldrich).

For PARP2 proteins, lysis was performed by sonication and the cell debris cleared by centrifugation (30,000*g* at 4 °C for 30 min). Supernatants were purified using HisPur Ni-NTA resin (Thermo Scientific) pre-equilibrated in lysis buffer. Resin was washed with wash 1 buffer (30 mM Hepes, 1 M NaCl, 10% glycerol, 10 mM imidazole, 0.5 mM TCEP, pH 7.5) and then with Wash 2 Buffer (30 mM Hepes, 500 mM NaCl, 10% glycerol, 25 mM imidazole, 0.5 mM TCEP, pH 7.5) before the elution step with elution buffer (30 mM Hepes, 300 mM NaCl, 10% glycerol, 200 mM imidazole, 0.5 mM TCEP, pH 7.5).

Eluted samples were then loaded to HiTrap Heparin column (GE healthcare) pre-equilibrated in Heparin A buffer (30 mM Hepes, 300 mM NaCl, 10% glycerol, 0.5 mM TCEP, pH 7.5) and washed with the same buffer. Proteins were eluted in a linear gradient to Heparin B buffer (30 mM Hepes, 1 M NaCl, 10% glycerol, 0.5 mM TCEP, pH 7.5 or 1.5 M for constructs in pNIC-Zb). Fractions containing protein were pooled and digested with TEV protease (1:30 TEV:PARP2 molar ratio) for 16 h at 4 °C. The samples were supplemented with 10 mM imidazole immediately before loading them to a 5 ml HisTrap column (GE Healthcare). The column was washed with Wash 2 Buffer and the flow through was concentrated in an Amicon ultra 15 device with 30 kDa cut-off. PARP2 protein samples were then further purified in a Superdex 75 pre-equilibrated in SEC buffer (30 mM Hepes, 350 mM NaCl, 10% glycerol, 0.5 mM TCEP, pH 7.5). Fractions containing PARP2 protein were pooled, concentrated, flash frozen and stored at −70 °C.

For HPF1, lysis was performed by sonication and in the presence of DNase I. The supernatant was filtered through a 0.45 µm filter and loaded onto a 5 ml HisTrap column (GE Healthcare). The column was washed with 180 ml of Wash 2 Buffer and eluted in elution buffer. The eluted sample was then diluted 1:5 in ion exchange loading buffer (25 mM Tris, 0.5 mM TCEP, pH 7.5) and loaded into a 5 ml HiTrap Q XL column (GE Healthcare) pre-equilibrated in ion exchange wash buffer (25 mM Tris, 100 mM NaCl, 0.5 mM TCEP, pH 7.5). The column was then washed with 50 ml of ion exchange wash buffer and eluted in a gradient from 100 mM to 1 M NaCl. The fusion tag was cleaved with TEV-protease at 4 °C for 16 h. 25 mM imidazole was added to the sample and then the protein was loaded onto a 5 ml HisTrap (GE Healthcare) and the flow through containing the cleaved proteins was collected, concentrated and further purified using Superdex 75 in SEC buffer. The purified protein was pooled, concentrated, flash frozen and stored in −70 °C.

The purified PARP2 and HPF1 proteins had absorbance ratios at 260 and 280 nm, 0.49–0.54 indicating that the protein batches did not contain nucleic acids that could affect the activity tests.

### CD

CD spectra of PARP2_FL_ WT, N127A, N129A, E286A, E286R and G338A were recorded at 22 °C using Chirascan CD spectroscopy (Applied Photophysics Ltd.) equipped with a temperature-regulated sample chamber. The far-UV region spectra (190–280 nm) were measured in a 1 mm path length quartz cuvette. The sample concentration was 0.05–0.07 mg/ml in 10 mM sodium phosphate pH 7.4, 150 mM (NH_4_)_2_SO_4_. The data were analysed with the Pro-Data Software suite (Applied Photophysics Ltd.).

### SDS-PAGE automodification activity assay

Automodification reactions containing 5 µg PARP2_FL_ and equimolar concentrations of HPF1 and 5′ phosphorylated hairpin oligonucleotide (DNA-3, see Supplementary Table 1) were initiated by the addition of 1 mM NAD^+^. The reaction buffer was 50 mM Tris, 5 mM MgCl_2_, pH 7.5. The samples were incubated at RT for 1 h and the reaction stopped by the addition of SDS containing buffer. The samples were then incubated 5 min at 95 °C and loaded onto a Mini-Protean 4–20% TGX gel (BioRad). Following electrophoresis, the gel was stained in PageBlue protein stain solution (Thermo Scientific).

### SEC-MALS

SEC-MALS analysis was performed as described previously^18^. Briefly, 35 µM PARP2_WGR+CAT_ and 37 µM DNA-1 were mixed in 20 mM HEPES pH 7.5, 400 mM NaCl, 0.5 mM TCEP and incubated for 1 h at RT before analysis. Samples were run in an S200 increase column (GE Healthcare) and analysed using the miniDAWN Treos II (Wyatt Technology). Mass determination was performed with ASTRA software (Wyatt Technology).

### Fluorescence activity assay

Activity assays of the PARP2 protein were done as reported earlier^17,18,38^. First, 50 nM of PARP2_FL_ WT or point mutant was mixed with 50 nM of each of the oligos (see Supplementary Table 1 for details) and 5 µM NAD^+^. The samples were then incubated at RT for 15 min for the mutant comparison assay and 1 h for the DNA-dependent activation assay. Measurements were done in quadruplicate and repeated 2 times. Conversion calculation was done in Microsoft Excel. IC_50_ determination was performed with 40 nM PARP2_FL_, 10 µg/ml activated DNA and 500 nM NAD^+^ and the reactions were incubated for 30 min at RT. IC_50_ measurements were done in quadruplicate and repeated 3 times. Data analysis was performed with an R script using the propagate package for first order Taylor expansion uncertainty estimation and nls function to fit the Hill equation.

### Fluorescence polarization

Fluorescence polarization was performed as previously described using fluorescein tagged dumbbell DNA containing a nick and 5′-phosphate or a double-stranded DNA model with 5′-phosphate (Supplementary Table 1)^17^. Measurements were done in triplicate and repeated 3 times.

### Crystallization

A 140 µl solution containing 150 μM PARP2_WGR-RD-ART_ and 160 μM DNA-1 (purchased from Integrated DNA Technology, IDT) was incubated on ice for 30 min. The complex was purified with size exclusion chromatography using a Superdex^TM^ S200 Increase 10/300 GL (GE-Healthcare) column pre-equilibrated with a buffer containing 20 mM Hepes, pH 7.5, 300 mM NaCl, 0.5 mM TCEP. The fractions corresponding to the complex were collected and concentrated for crystallization. The crystallization was done using a sitting drop vapour diffusion method at +4 °C and the precipitant solution was 0.1 M MES pH 6.5 and 1 M ammonium sulfate. Notably, successful crystallization required optimization of the DNA length. Our previous studies showed that the catalytic activity of PARP2 at a physiological salt concentration of 150 mM was higher when DNAs with lengths of 10–20 base pairs (bp) were used^17^. Based on this information, we started crystallization experiments of PARP2_WGR-RD-CAT_ with DNAs within this range. We observed that crystallization of PARP2_WGR-RD-CAT_ with 12 and 15 bp DNAs produced only micro-crystals of less than 5 µm, while crystallization with 16 and 20 bp DNAs produced crystals which were large enough for data collection. However, the crystals with 20 bp DNA were very fragile and diffracted only to 8 Å in the initial data collection using the X-ray diffractometer with a rotating anode at Biocenter Oulu, Finland. However, the crystals with 16 bp DNA had much better diffraction, 4 Å, suggesting that the 16 bp dsDNA phosphorylated at one of the 5′ ends (DNA-1; Supplementary Table 1) offered a better crystal packing.

### Data collection, structure determination and refinement

In initial data collections at synchrotrons, we noticed that the diffraction quality of the crystals of the PARP2_WGR-RD-ART_-DNA-1 complex was highly dependent on cryo-protectants. In addition, the crystals suffered greatly from radiation damage and the diffraction was nonuniform requiring a grid scan to locate the best diffracting positions of a single crystal. We tested PEG400 and glycerol supplemented with the mother liquor (0.1 M MES pH 6.5, 1 M ammonium sulfate) as cryo-solutions, but neither of them worked well, as the crystals had a modest diffraction to 4 Å. By combining different cryo-protectants together, the diffraction quality and resolution were improved. Finally, we used 0.1 M MES pH 6.5, 1 M ammonium sulfate, 10% (v/v) glycerol, 10% (v/v) diethylene glycol and 10% (v/v) 2-propanol as a cryo-solution. However, the crystals still suffered significantly from radiation damage allowing only collection of approximately 10˚ oscillation with a resolution between 2.7 and 3 Å. This together with nonuniform diffraction made it impossible to obtain a complete dataset from a single crystal. In order to overcome the problem, we located the best diffracting positions of multiple crystals using grid scans and collected multiple small datasets from the positions on the beamline i24 at the Diamond Light Source (UK). The datasets were processed using XDS^39^ and their correlation in terms of unit cells and space group were analysed using the ccCluster program^40^. Visual analysis of the dendogram in ccCluster GUI showed that the majority of the datasets were identical within a 0.2 threshold. Finally, we selected 26 datasets collected from 4 crystals for merging that yielded good statistics with a resolution of 2.8 Å in XSCALE^39^. CC1/2 was used as a high-resolution cut-off criterion^41^. The data collection and structure refinement statistics are presented in Table 1.

Phases for the PARP2_WGR-RD-ART_-DNA-1 complex structure were solved using molecular replacement with several cycles. First, the PARP2 WGR and ART domain structures (PDB id. F61K and 5DSY, respectively)^18,24^ together were used as search models in MRBUMP^42^ included with the protein and DNA sequences. As a result, we obtained an incomplete solution where only the WGR domain and DNA-1 were correctly placed. Next, we performed a second run using the incomplete solution and the ART domain (PDB id. 5DSY) as a search model, and obtained a complete solution where the ART domain was correctly placed together with the WGR domain and DNA-1. The structure was initially refined with REFMAC5^43^ and we were able to trace the C-terminus of the WGR domain and the N-terminus of the ART domain from the structure allowing us to manually build the missing RD domain using Coot^44^. Finally, the PARP2_WGR-RD-ART_ DNA-1 complex structure was refined with Phenix^45^. The data collection and refinement statistics are presented in Table 1. The figures of the structures were made using Pymol^46^.

### Reporting summary

Further information on research design is available in the Nature Research Reporting Summary linked to this article.

## Supplementary information

Supplementary information.

Description of Additional Supplementary Files.

Supplementary Movie 1.

Reporting summary.

## Data Availability

The data that support the findings of this study are available from the corresponding author upon reasonable request. Atomic coordinates and structure factors have been deposited to the Protein Data Bank under accession number 7AEO and raw diffraction images are available via Zenodo. Previously published crystal structures used to derive the models shown are F61K^18^, 5DSY^24^, 4TVJ^36^, 4DQY^15^, 6BHV^25^, 6TX3^33^ and 1A26^37^. Source data are provided with this paper.

## Source data

Source Data.
