# Supplementary material for: Activation of PARP2/ARTD2 by DNA damage induces conformational changes relieving enzyme autoinhibition
Source: Nat Commun. 2021 Jun 9;12:3479. doi: 10.1038/s41467-021-23800-x (PMC8190142; doi:10.1038/s41467-021-23800-x)
Supplement: Supplementary file 1 — Supplementary information. [file 41467_2021_23800_MOESM1_ESM.pdf]

Supplementary Information

Activation of PARP2/ARTD2 by DNA damage induces conformational changes relieving enzyme autoinhibition

Ezeogo Obaji[1], Mirko M. Maksimainen[1], Albert Galera-Prat[1] & Lari Lehtiö[1]*.

Affiliations:
[1]Faculty of Biochemistry and Molecular Medicine & Biocenter Oulu, University of Oulu, Oulu, Finland.

*Corresponding author: Lari Lehtiö, e-mail: lari.lehtio@oulu.fi

Content:

Supplementary Table 1. Oligonucleotides used in the binding studies.
Supplementary Table 2. Primers used in the study.
Supplementary Fig. 1. Representative electron density map.
Supplementary Fig. 2. SEC-MALS of $PARP2_{WGR-RD-ART}$ DNA-1 complex.
Supplementary Fig. 3. Thymine binding to the active site.
Supplementary Fig. 4. PARP2 inhibition by thymine derivatives and activation by DNA models.
Supplementary Fig. 5. PARP2 affinity to DNA models.
Supplementary Fig. 6. CD spectra of $PARP2_{FL}$ wt and mutants.
Supplementary Fig. 7. Activated PARP2 model is compatible with HPF1 binding.

Supplementary Table 1. Oligonucleotide sequences used in this study.

Schematic	Oligonucleotides	Sequence
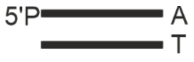	DNA-1	Forward: 5’phosphate GAC GAC CCG GAG CAC A 3’<br>Reverse: 3’ TGT GCT CCG GGT CGT C 3’
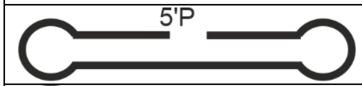	DNA-2	5'phosphate GGG TCT TTT GAC CCT CGA GCT TTT GCT CGA 3’
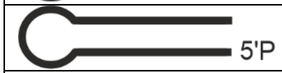	DNA-3	5'phosphate GCC TAG CTA CGT AGC TAG GC 3’
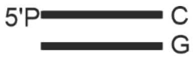	DNA-4	Forward: 5’phosphate GAC GAC CCG GAG CAC C 3’<br>Reverse: 5’ GGT GCT CCG GGT CGT C 3’
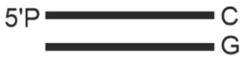	DNA-5	Forward: 5’phosphate GAC GCA ACC CGG AGA ACA CC 3’<br>Reverse: 5’GGT GTT CTC CGG TTG CGT C 3’
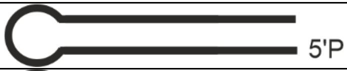	DNA-6	5’phosphate GCC TAG CTA CGT AGC TAG GC 3’
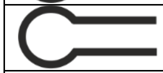	DNA-7	5’ GCC TAT ATA GGC 3’
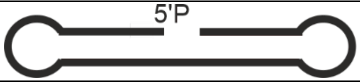	DNA-8	5’phosphate GGA AGT CTT T(Fluorescein) TGA CTT CCT CGA AGC TTT TGC TTC GA 3’
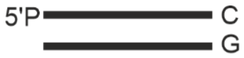	DNA-9	5´phosphate GAC GCA ACC CGG AGA ACA CA 3’<br>Reverse: 5’ TGT GTT CT(Fluorescein)C CGG GTT GCG TC 3’

Supplementary Table 2. Primers used for cloning the constructs and the mutants generated in this study.

Primer	Sequence
P2_FL_fwd	GGTACCGAGAACCTGTACTTCCAATCC
P2_G90_fwd	GGTACCGAGAACCTGTACTTCCAATCCATATCAATCACTGAAAAAG
P2_FL_rev	GATCCGTATCCACCTTTACTGGAGACC
P2_N129A_fwd	CGAACCTGCAATTCAACAACGCCAAGTACTACCTGATCCAGC
P2_N129A_rev	GCTGGATCAGGTAGTACTTGGCGTTGTTGAATTGCAGGTTCG
P2_E286A_fwd	ATCACTGAAAAAGATCGCAGACTGCATTCGTGCCG
P2_E286A_rev	CGGCACGAATGCAGTCTGCGATCTTTTTCAGTGAT
P2_E286R_fwd	ATCAATCACTGAAAAAGATCAGAGACTGCATTCGTGCCGGTC
P2_E286R_rev	GACCGGCACGAATGCAGTCTCTGATCTTTTTCAGTGATTGAT
P2_G338A_fwd	GCGATTTCGATATCGGCCAGTGCTTCCAGCAGTTGG
P2_G338A_rev	CCAACTGCTGGAAGCACTGGCCGATATCGAAATCGC
HPF1_fwd	GGTACCGAGAACCTGTACTTCCAATCCATGGTGGGTGGCGGTGGCAAAC
HPF1_rev	GATCCGTATCCACCTTTACTGGAGACCGTCTTACGCCGCCAGTTGATCAATGTTCTC

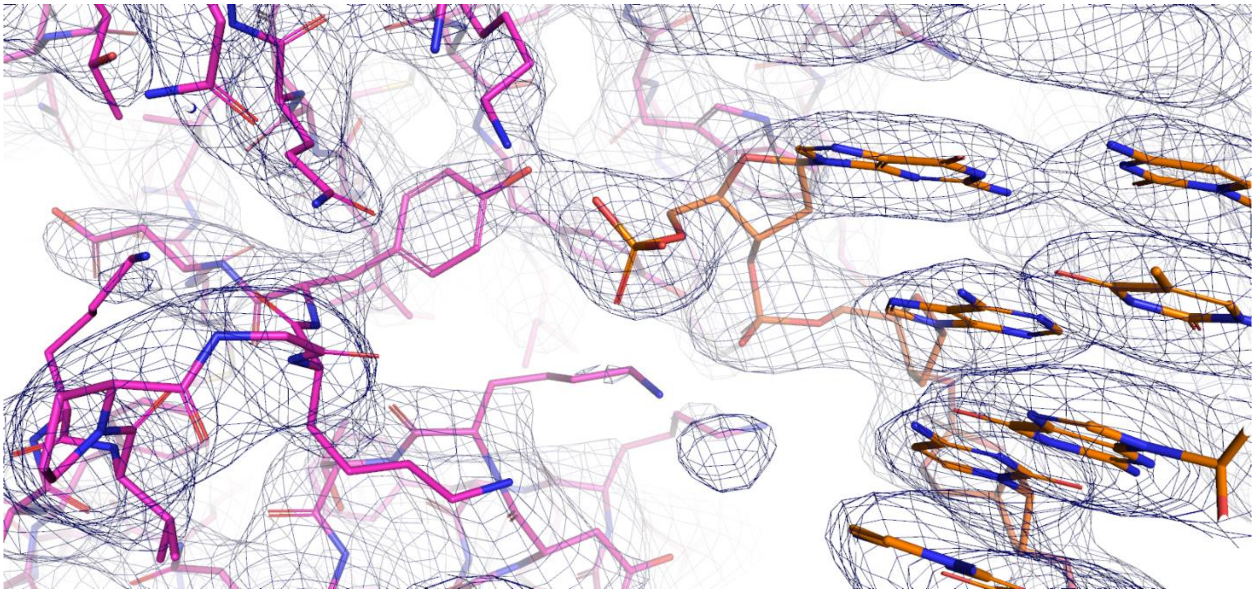

Supplementary Fig. 1. Representative picture of an electron density map. Sigma A weighted 2Fo-Fc map is contoured at 1 σ at the DNA damage recognition site.

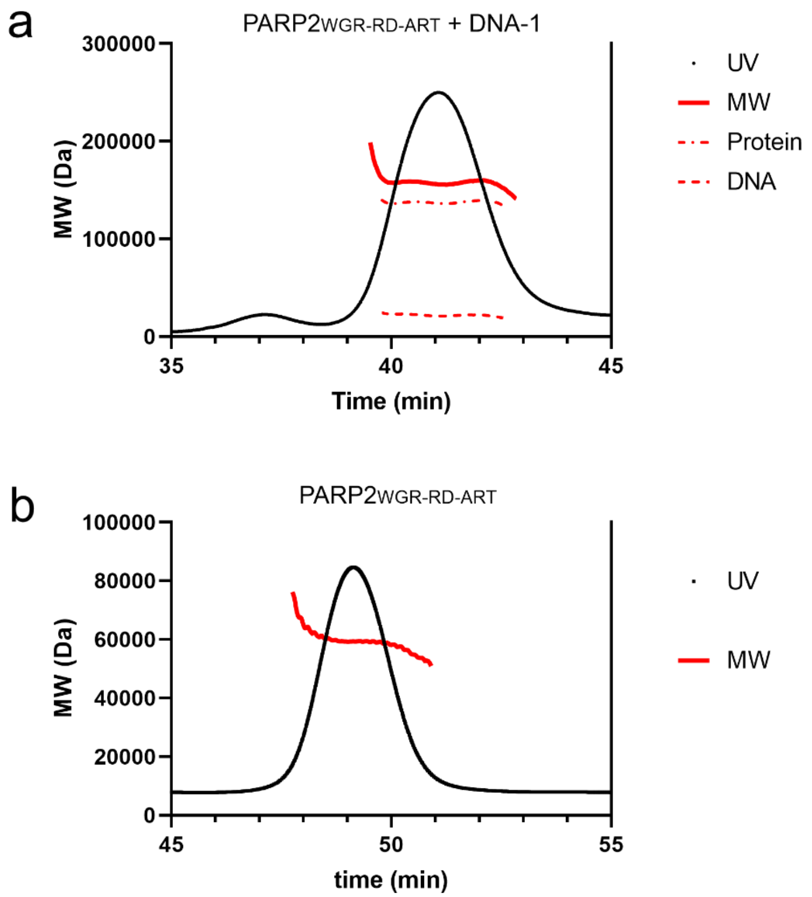

Supplementary Fig. 2. SEC-MALS of PARP2$_{WGR-RD-ART}$ DNA-1 complex. (**a**) 35 µM of PARP2$_{WGR-RD-ART}$ was mixed with 37 µM of DNA-1 and the complex was analyzed using SEC-MALS. The experimentally determined MW was 158 ± 3 kDa. Protein conjugate analysis indicated that the protein fraction contribution would be 137 ± 2 kDa while the DNA was 21 ± 3 kDa. This would correspond to a complex observed in the crystal although the measured protein MW is larger corresponding to 2.4:2 protein:DNA ratio. (**b**) SEC-MALS analysis of PARP2$_{WGR-RD-ART}$ without DNA. The measured molecular weight (59.4 ± 0.7 kDa) confirms that protein is a monomer (theoretical molecular weight 56.6 kDa). Source data are provided as a Source Data file.

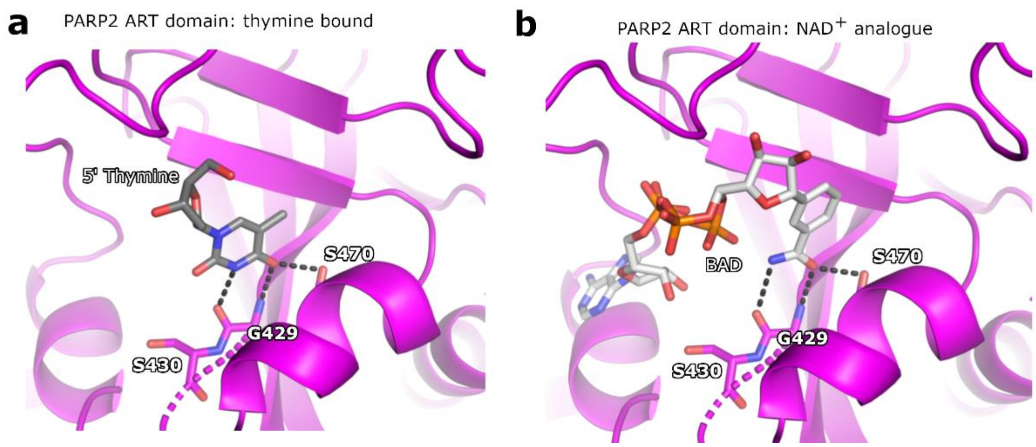

Supplementary Fig. 3. (**a**) Close up view of the 5´-thymidine in the nicotinamide binding site**.** (**b**) Binding of an NAD$^+$ analog to the active site superimposed from the PARP1 complex structure (PDB id. 6BHV).

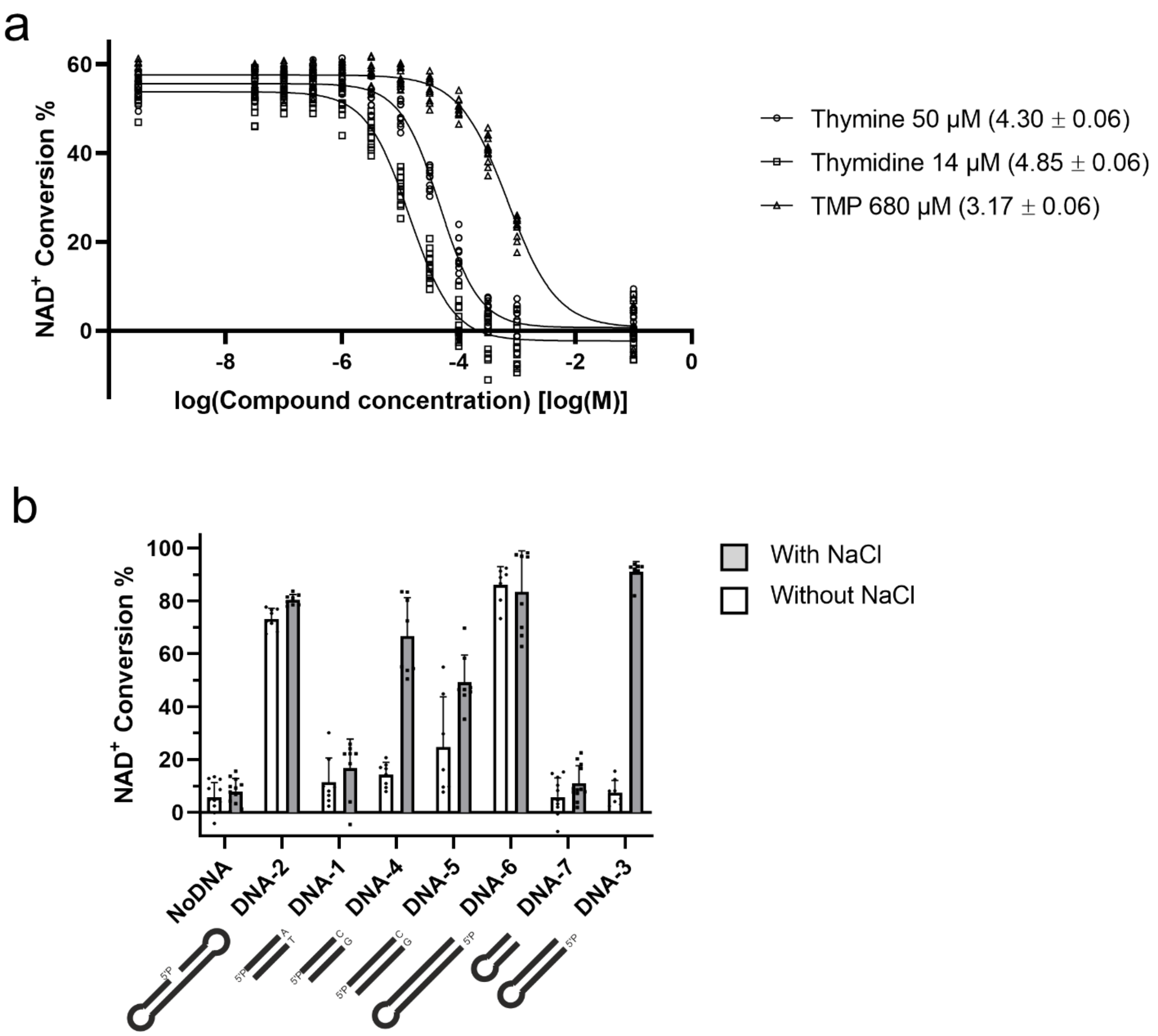

Supplementary Fig. 4. PARP2 activation by DNA. (**a**) IC_{50} determination of Thymine, Thymidine and TMP with $PARP2_{FL}$. Individual data points obtained from 3 independent experiments are shown. Calculated values correspond to IC_{50} (pIC_{50} ± S.D.) (**b**) Catalytic activity of PARP2 in the presence of the oligonucleotide used in crystallization (DNA-1) is very low. Replacement of the terminal A-T pair on DNA-1 with C-G (DNA-4) as well as longer double-stranded DNA terminated in C-G (DNA-5) promote activation of PARP2 in the absence of NaCl. 5´-phosphorylated dumbbell and hairpin DNA (DNA-2, DNA-3 and DNA-6) used as positive controls and non-phosphorylated DNA-7 used as negative control. Data shows individual conversion values obtained from 2 independent experiments. Bars represent the average of those values and error bar corresponds to standard deviation. Source data are provided as a Source Data file.

a

	WT	N129A	R153A	Y201F	E286A	E286R	G338A
**DNA-8**	8.9 ± 1.6	9.6 ± 4.4	13.0 ± 6.3	41.0 ± 11	8.1 ± 2.4	10.6 ± 7.3	9.0 ± 2.3
**DNA-9**	25 ± 3	48 ± 22	153 ± 85	97 ± 52	24 ± 2	18 ± 6	28 ± 2

b

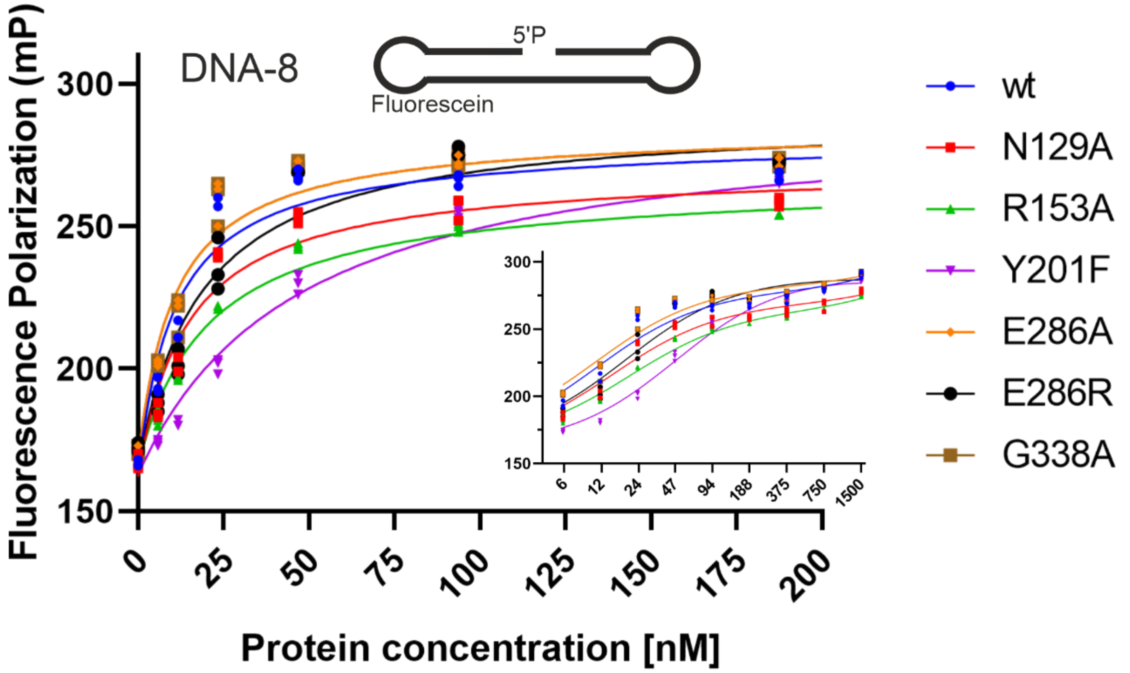

c

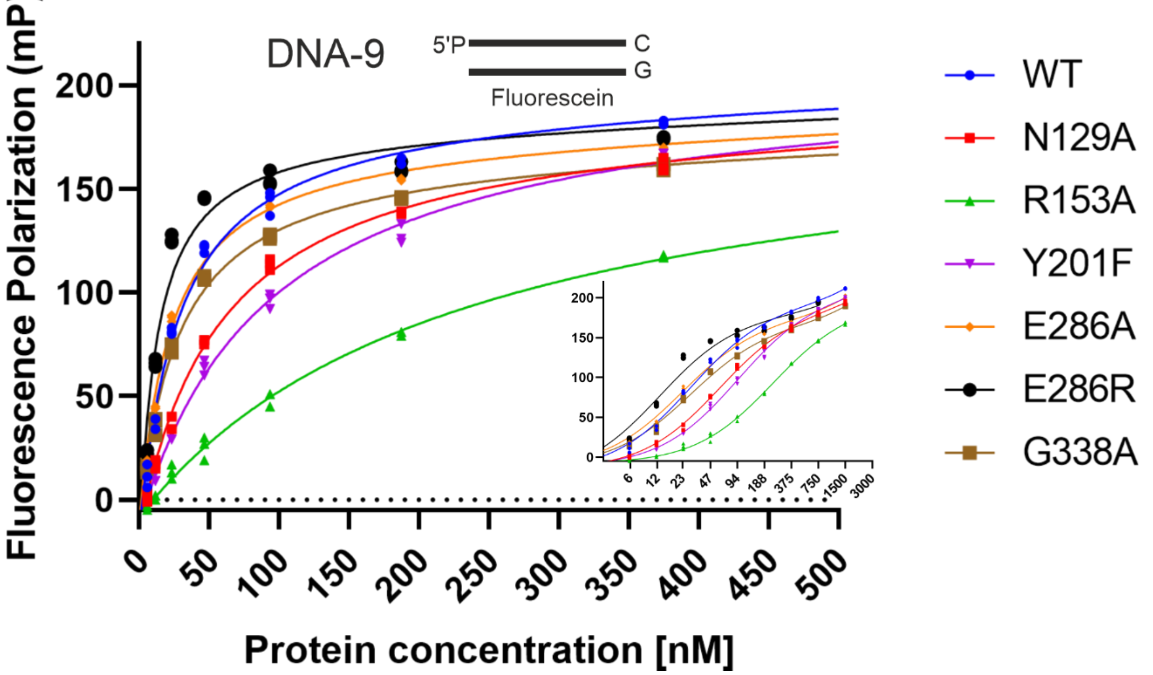

Supplementary Fig. 5. Fluorescence polarization assay to determine the affinity of $PARP2_{FL}$ mutants to DNA. (**a**) Estimated K_D in nM. Values correspond to average ± s.d. of 3 independent

experiments each done with triplicates. (**b**-**c**) Example curves of $PARP2_{FL}$ mutants titration against dumbbell nicked and 5'-phosphorylated DNA (DNA-8, **b**) and 5'-phosphorylated double-stranded blunt end DNA (DNA-9, **c**). Data points representing individual measurements. Insets show all the points used in each fitting in log2 scale for clarity in the low concentration area. Data was fitted with Graphpad Prism. Source data are provided as a Source Data file.

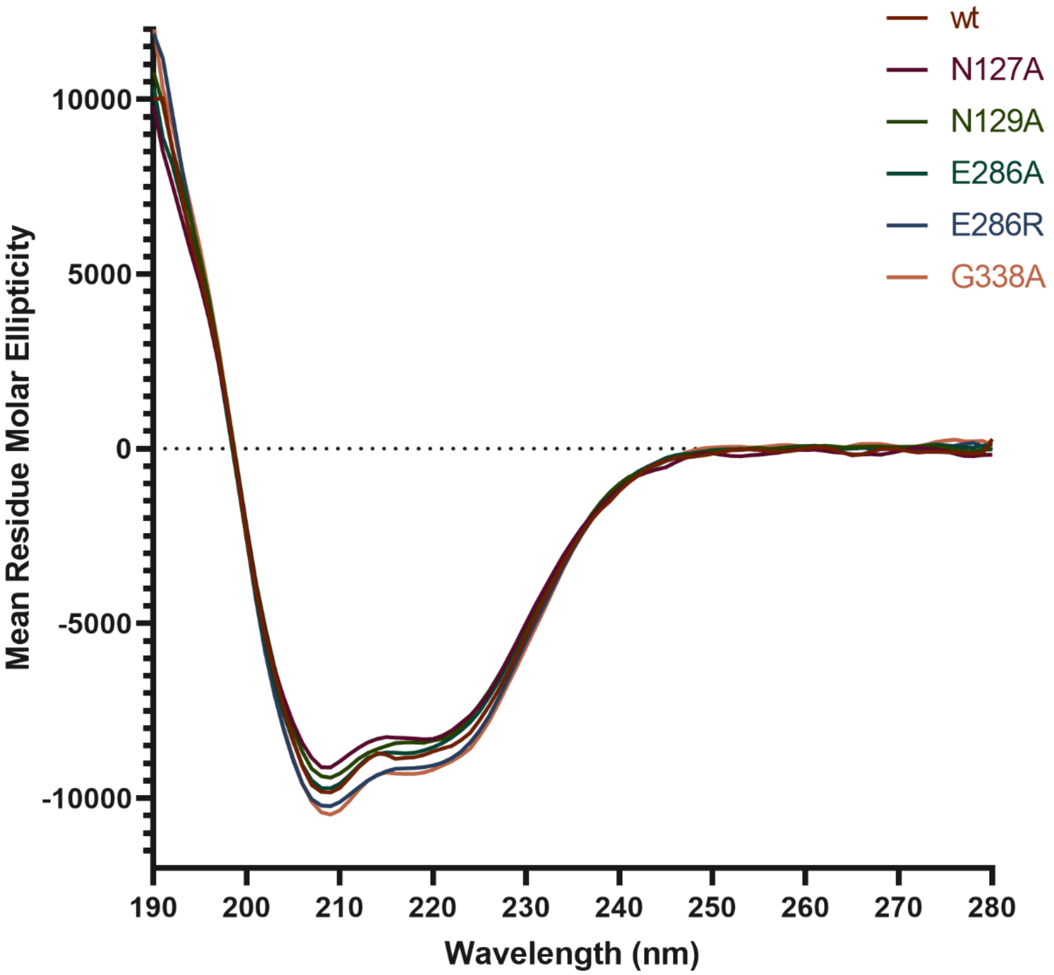

Supplementary Fig. 6. CD spectra of PARP2$_{FL}$ wt and mutants. Source data are provided as a Source Data file.

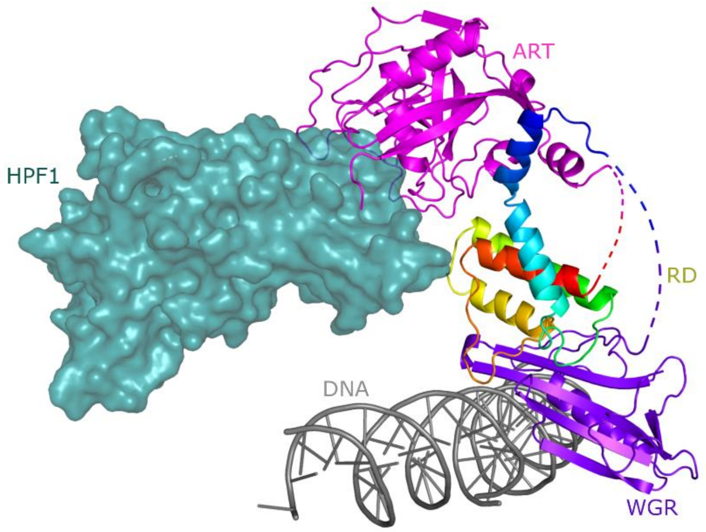

Supplementary Fig. 7. Modelled PARP2 crystal structure-HPF1 complex. PARP2 crystal structure in the activated state was modelled in complex with HPF1 (PDB id. 6TX3).
