# Supplementary material for: Activation of PARP2/ARTD2 by DNA damage induces conformational changes relieving enzyme autoinhibition
Source: Nat Commun. 2021 Jun 9;12:3479. doi: 10.1038/s41467-021-23800-x (PMC8190142; doi:10.1038/s41467-021-23800-x)
Supplement: Supplementary file 2 — Description of Additional Supplementary Files. [file 41467_2021_23800_MOESM2_ESM.docx]

**Description of Supplementary Files**

**File Name: Supplementary Movie 1**

**Description:** A movie showing structural changes upon activation of PARP2 and subsequent binding of substrate NAD+ and other proteins during the reaction cycle. PDB codes used to derive the models are indicated within the movie.
