# Supplementary material for: Activation of PARP2/ARTD2 by DNA damage induces conformational changes relieving enzyme autoinhibition
Source: Nat Commun. 2021 Jun 9;12:3479. doi: 10.1038/s41467-021-23800-x (PMC8190142; doi:10.1038/s41467-021-23800-x)
Supplement: Supplementary file 4 — Reporting summary. [file 41467_2021_23800_MOESM4_ESM.pdf]

nature research

Corresponding author(s): Lari Lehtiö

Last updated by author(s): May 10, 2021

Reporting Summary

Nature Research wishes to improve the reproducibility of the work that we publish. This form provides structure for consistency and transparency in reporting. For further information on Nature Research policies, see our Editorial Policies and the Editorial Policy Checklist.

Statistics

For all statistical analyses, confirm that the following items are present in the figure legend, table legend, main text, or Methods section.

n/a	Confirmed	
☐	☒	The exact sample size (*n*) for each experimental group/condition, given as a discrete number and unit of measurement
☒	☐	A statement on whether measurements were taken from distinct samples or whether the same sample was measured repeatedly
☒	☐	The statistical test(s) used AND whether they are one- or two-sided<br>*Only common tests should be described solely by name; describe more complex techniques in the Methods section.*
☒	☐	A description of all covariates tested
☒	☐	A description of any assumptions or corrections, such as tests of normality and adjustment for multiple comparisons
☒	☐	A full description of the statistical parameters including central tendency (e.g. means) or other basic estimates (e.g. regression coefficient) AND variation (e.g. standard deviation) or associated estimates of uncertainty (e.g. confidence intervals)
☒	☐	For null hypothesis testing, the test statistic (e.g. *F*, *t*, *r*) with confidence intervals, effect sizes, degrees of freedom and *P* value noted<br>*Give P values as exact values whenever suitable.*
☒	☐	For Bayesian analysis, information on the choice of priors and Markov chain Monte Carlo settings
☒	☐	For hierarchical and complex designs, identification of the appropriate level for tests and full reporting of outcomes
☒	☐	Estimates of effect sizes (e.g. Cohen's *d*, Pearson's *r*), indicating how they were calculated

Our web collection on statistics for biologists contains articles on many of the points above.

Software and code

Policy information about availability of computer code

Data collection	Diffraction data was collected using a GDA beamline i24 software at Diamond Light Source. CD data was collected using Chirascan 4.5.1848.0 (Wyatt Technology).
Data analysis	Diffraction data was analysed with ccCluster (0.2) and processed with XDS (VERSION Mar 15, 2019 BUILT=20190315). Structure was refined using Refmac5 (5.8.0253) and Phenix (1.11.1). Model was built with Coot (0.9.2) and visualized with Pymol (2.3.0). MALS data was analyzed with ASTRA software 7.3.2 (Wyatt Technology). Fluorescence polarization data was fitted Graphpad Prism 8. CD data was analyze with Pro-Data Software suite 4.2.15 (Applied Photophysics Ltd.). Data analysis for IC50 measurements was performed with a R (3.6.3) script using the propagate package for first order Taylor expansion uncertainty estimation and nls function to fit the Hill equation. No custom algorithms or software were used in the data analysis.

For manuscripts utilizing custom algorithms or software that are central to the research but not yet described in published literature, software must be made available to editors and reviewers. We strongly encourage code deposition in a community repository (e.g. GitHub). See the Nature Research guidelines for submitting code & software for further information.

Data

Policy information about availability of data

All manuscripts must include a data availability statement. This statement should provide the following information, where applicable:

- Accession codes, unique identifiers, or web links for publicly available datasets
- A list of figures that have associated raw data
- A description of any restrictions on data availability

The data that support the findings of this study are available from the corresponding author upon reasonable request. Source data for biochemical and biophysical assays are provided with this paper. Atomic coordinates and structure factors have been deposited to the Protein Data Bank under accession number 7AEO [http://

doi.org/10.2210/pdb7AEO/pdb] and raw diffraction images are available at Zenodo [http://doi.org/10.5281/zenodo.4449849]. Previously published crystal structures used to derive the models shown are F61K [https://www.rcsb.org/structure/6F1K/pdb]18, 5DSY [https://www.rcsb.org/structure/5DSY/pdb]24, 4TVJ [http://doi.org/10.2210/pdb4TVJ/pdb]36, 4DQY [http://doi.org/10.2210/pdb4DQY/pdb]15, 6BHV [http://doi.org/10.2210/pdb6BHV/pdb]25, 6TX3 [http://doi.org/10.2210/pdb6TX3/pdb]33 and 1A26 [http://doi.org/10.2210/pdb1A26/pdb]37.

Field-specific reporting

Please select the one below that is the best fit for your research. If you are not sure, read the appropriate sections before making your selection.

☒ Life sciences ☐ Behavioural & social sciences ☐ Ecological, evolutionary & environmental sciences

For a reference copy of the document with all sections, see nature.com/documents/nr-reporting-summary-flat.pdf

Life sciences study design

All studies must disclose on these points even when the disclosure is negative.

Sample size	Test set reflection set for Rfree calculation was set to 4.7% (1175 random reflections) that is sufficient for validation of the refinement according to published best practices in the field (Kleywegt & Brünger (1996) Structure 4:897-904).
Data exclusions	Data was not excluded with an exception of clear experimental outlier (single concentration in FP measurements).
Replication	Data for IC50s were measured in quadruplicates and measurement was repeated three times. Kd measurements were carried out in triplicates and repeated three times. Gel activity assay has been repeated once with similar results and multiple times for the wt enzyme. All replications were successful.
Randomization	Only randomization carried out during the studies was the generation of a test of reflections for the calculation of an Rfree factor for refinement validation.
Blinding	The study does not include clinical studies or similar requiring blinding. Structural studies use our prior knowledge of macromolecule structures to allow restrained refinement, but the bias created is a prerequisite and limited.

Reporting for specific materials, systems and methods

We require information from authors about some types of materials, experimental systems and methods used in many studies. Here, indicate whether each material, system or method listed is relevant to your study. If you are not sure if a list item applies to your research, read the appropriate section before selecting a response.

Materials & experimental systems

n/a	Involved in the study	
☒	☐	Antibodies
☒	☐	Eukaryotic cell lines
☒	☐	Palaeontology and archaeology
☒	☐	Animals and other organisms
☒	☐	Human research participants
☒	☐	Clinical data
☒	☐	Dual use research of concern

Methods

n/a	Involved in the study	
☒	☐	ChIP-seq
☒	☐	Flow cytometry
☒	☐	MRI-based neuroimaging
